# Biofilm-Forming Methicillin-Resistant Staphylococcus aureus: A Comprehensive Phenotypic and Genotypic Review

**DOI:** 10.7759/cureus.108813

**Published:** 2026-05-13

**Authors:** Sejal R Bhujugade, Harsha V Patil, Satish R Patil

**Affiliations:** 1 Department of Microbiology, Krishna Institute of Medical Sciences, Krishna Vishwa Vidyapeeth (Deemed to be University), Karad, IND

**Keywords:** agr system, antimicrobial resistance, biofilm, genotypic analysis, ica operon, mrsa, phenotypic detection, sara

## Abstract

Methicillin-resistant *Staphylococcus aureus* (MRSA) is a major global pathogen, capable of forming biofilms that confer enhanced antimicrobial tolerance, virulence, and persistence in both hospital and community settings. Biofilms are structured microbial communities encased in extracellular polymeric substances, composed of polysaccharides, proteins, and extracellular DNA. Complex genetic networks, including the ica operon, the agr quorum-sensing system, the sarA regulatory system, and stress-response genes such as sigB, regulate MRSA biofilm formation. Phenotypic methods (Congo red agar, microtiter plate, and tube test) and genotypic assays (PCR-based detection of ica, fnbA/B, clfA/B, sarA, and agr) provide insights into biofilm capacity and virulence potential. Clinically, MRSA biofilms contribute to device-related infections, chronic wounds, and recurrent infections, challenging conventional therapies such as vancomycin, daptomycin, and rifampicin. Emerging strategies, including enzymatic matrix degradation, bacteriophage therapy, quorum-sensing inhibitors, and nanoparticle-based drug delivery, offer potential alternatives. This review synthesizes current knowledge on MRSA biofilm phenotypes and genotypes, highlighting molecular mechanisms, clinical significance, and therapeutic approaches.

## Introduction and background

*Staphylococcus aureus* is a Gram-positive, facultative anaerobe widely distributed on human skin and mucous membranes. While often a commensal organism, it is an opportunistic pathogen responsible for superficial skin infections, abscesses, and severe invasive diseases such as bacteremia, endocarditis, osteomyelitis, and pneumonia [1,2]. The emergence of methicillin-resistant *Staphylococcus aureus* (MRSA) in the 1960s marked a significant turning point in the history of antimicrobial resistance. MRSA strains harbor the mecA gene, located on the staphylococcal cassette chromosome mec, which encodes penicillin-binding protein 2a, conferring resistance to all β-lactam antibiotics [3]. Additional resistance determinants frequently render these strains multidrug-resistant, complicating treatment [4,5].

Biofilm formation is a critical factor contributing to MRSA persistence and virulence [6]. Biofilms are organized communities of bacteria embedded within a self-produced extracellular polymeric substance (EPS), composed of polysaccharides, proteins, and extracellular DNA (eDNA) [7]. The biofilm lifestyle protects MRSA from host immune responses and significantly reduces the efficacy of antibiotics, often requiring concentrations up to 1,000-fold higher than those needed to eradicate planktonic cells [8,9]. Moreover, biofilms facilitate horizontal gene transfer, phenotypic heterogeneity, and activation of stress responses, collectively enhancing bacterial survival under adverse conditions [10,11].

Recent studies indicate that phenotypic differences between methicillin-sensitive *Staphylococcus aureus* (MSSA) and MRSA biofilms are primarily related to matrix composition and regulatory pathways rather than methicillin resistance alone. MSSA biofilms are typically ica-dependent and polysaccharide-rich, driven by polysaccharide intercellular adhesin (PIA) synthesis via the icaADBC operon. In contrast, MRSA biofilms are more often ica-independent, relying on eDNA, surface-associated proteins (e.g., LPXTG-anchored adhesins and SDR proteins), and autolysis-mediated matrix release, resulting in a more proteinaceous and heterogeneous structure. These compositional differences contribute to phenotypic traits such as increased structural adaptability, immune evasion, and antimicrobial tolerance in MRSA biofilms [12]. However, comparative studies show inconsistent differences in total biofilm biomass: some report no significant difference in biofilm-forming capacity between MSSA and MRSA clinical isolates, with both frequently exhibiting strong biofilm phenotypes [13], while others emphasize that strain background, regulatory systems (e.g., agr, sarA), and environmental conditions are more determinative than resistance status itself [14]. Collectively, current evidence supports the view that MSSA and MRSA differ in biofilm architecture and matrix dependency, but not necessarily in overall biofilm-forming ability, highlighting the multifactorial nature of *Staphylococcus aureus* biofilm phenotypes.

Understanding MRSA biofilm biology, including molecular regulatory pathways and both phenotypic and genotypic detection methods, is essential for designing targeted therapeutic strategies. This review provides a comprehensive overview of MRSA biofilm formation, regulation, clinical impact, detection techniques, and emerging anti-biofilm interventions, emphasizing open-access, contemporary research.

Methods

A comprehensive literature search was conducted to identify relevant studies on biofilm-forming MRSA. Electronic databases, including PubMed, Scopus, Web of Science, and Google Scholar, were systematically searched for articles published between 2010 and 2025. The search strategy used combinations of keywords and Boolean operators, including “MRSA,” “biofilm formation,” “methicillin-resistant *Staphylococcus aureus*,” “ica operon,” “biofilm-associated genes,” “phenotypic detection,” “genotypic characterization,” and “antimicrobial resistance.”

Only peer-reviewed articles published in English were included. Studies focusing on clinical isolates, molecular mechanisms of biofilm formation, phenotypic and genotypic characterization, antibiotic resistance, and therapeutic approaches were considered eligible. Duplicate publications, conference abstracts without full text, non-English publications, and studies lacking adequate methodological details were excluded.

Relevant references from selected articles were also manually screened to identify additional studies. The retrieved literature was critically analyzed to summarize current evidence regarding the phenotypic and genotypic determinants of biofilm formation in MRSA and their clinical implications.

## Review

Biofilm formation and matrix composition

Biofilm formation in MRSA is a highly regulated, dynamic, and multistage process that enables bacterial persistence in both host tissues and on abiotic surfaces such as medical devices. This process is typically divided into four sequential but overlapping stages: adhesion, accumulation, maturation, and dispersal [6,7]. MRSA strains exhibit remarkable heterogeneity in biofilm-forming capacity, capable of producing both PIA-dependent and PIA-independent biofilms enriched in proteins and eDNA. The predominance of each mechanism is strain-specific and influenced by environmental conditions and global regulatory systems, including agr, sarA, and sigB.

The initial stage of biofilm formation, adhesion, involves the attachment of planktonic MRSA cells to biotic or abiotic surfaces. This step is mediated primarily by a class of surface proteins known as microbial surface components recognizing adhesive matrix molecules (MSCRAMMs). These adhesins facilitate binding to host extracellular matrix components, including fibronectin, fibrinogen, collagen, and elastin. Key MSCRAMMs include fibronectin-binding proteins (fnbA and fnbB), clumping factors (clfA and clfB), and collagen-binding adhesins [6].

In addition to protein-mediated attachment, physicochemical interactions such as hydrophobicity, electrostatic forces, and surface conditioning films (e.g., plasma proteins deposited on medical implants) further enhance adhesion. This stage is critical for successful colonization, particularly in clinical settings where MRSA adheres to indwelling devices like catheters, prosthetic joints, and cardiac implants. Once attached, bacterial cells undergo phenotypic changes that promote irreversible adherence and lead to the transition to the next stage.

Following initial adhesion, MRSA cells proliferate and form multilayered clusters via intercellular adhesion mechanisms. In many strains, this process is mediated by PIA, a β-1,6-linked N-acetylglucosamine polymer synthesized by enzymes encoded by the icaADBC operon. PIA promotes cell-to-cell adhesion and contributes to immune evasion by masking bacterial surface antigens.

However, not all MRSA biofilms are PIA-dependent. In PIA-independent biofilms, accumulation is driven by surface-associated and secreted proteins, including accumulation-associated protein (Aap), biofilm-associated protein (Bap), and extracellular adherence protein (Eap). Additionally, eDNA, released through controlled autolysis mediated by enzymes such as autolysin (Atl), plays a crucial structural role by forming a scaffold that stabilizes the biofilm matrix [7,10].

The relative contribution of PIA, proteins, and eDNA varies with the strain's genetic background and environmental factors, including nutrient availability, osmolarity, and antibiotic exposure. This compositional variability contributes to differences in biofilm architecture, mechanical stability, and resistance to antimicrobial agents.

During the maturation stage, MRSA biofilms develop into complex, three-dimensional structures characterized by architectural heterogeneity and functional specialization. The biofilm matrix expands to form multilayered cell clusters embedded within an EPS, creating a highly organized community. Within this structure, gradients of nutrients, oxygen, pH, and metabolic byproducts are established, resulting in distinct microenvironments that influence bacterial physiology.

These gradients lead to metabolic heterogeneity, where cells in the outer layers remain metabolically active, while those in deeper layers often adopt a slow-growing or dormant phenotype. This physiological diversity enhances overall biofilm resilience, particularly against antimicrobial agents, as dormant cells are less susceptible to antibiotics that target active cellular processes.

Additionally, water channels form within the biofilm, functioning as primitive circulatory systems that facilitate the distribution of nutrients and removal of waste products. This organized architecture promotes cooperative interactions among bacterial cells, including quorum sensing, metabolic cross-feeding, and stress adaptation. Collectively, these features contribute to the enhanced survival, persistence, and chronicity of MRSA infections [6,7].

Dispersal represents the final stage of the biofilm lifecycle, during which bacterial cells detach from the mature biofilm and return to a planktonic state, enabling colonization of new niches. This process can occur through both passive mechanisms (e.g., shear forces, fluid flow) and active, regulated pathways.

Active dispersal is primarily mediated by enzymatic degradation of the biofilm matrix. MRSA produces several enzymes, including proteases and nucleases, as well as surfactant-like molecules, such as phenol-soluble modulins, that break down key matrix components, including proteins and eDNA. This degradation weakens the biofilm's structural integrity, facilitating cell release.

A central regulator of dispersal is the agr quorum-sensing system. Activation of the agr system leads to the upregulation of virulence factors and matrix-degrading enzymes, while simultaneously downregulating surface adhesins. This shift promotes detachment and dissemination of bacterial cells. Importantly, agr activity is often suppressed during early biofilm formation and becomes upregulated during later stages, highlighting its role in biofilm turnover and the spread of infection.

**Figure 1:**
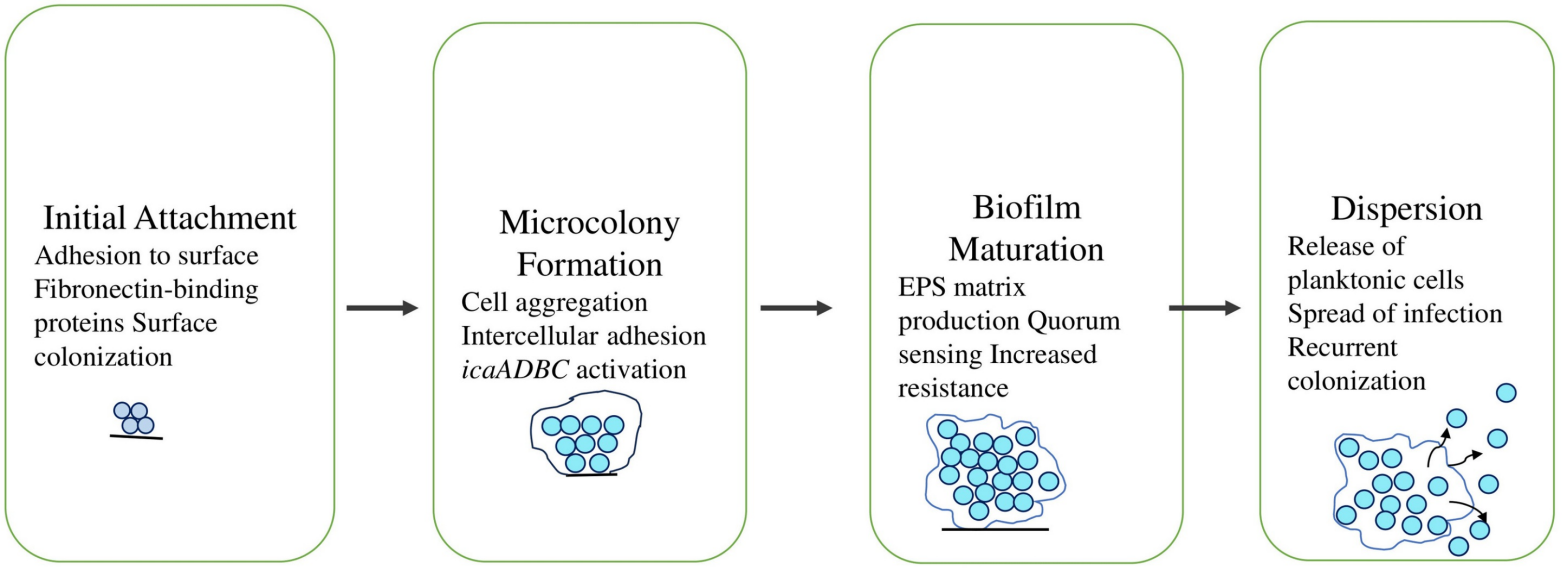
Sequential stages involved in MRSA biofilm formation including attachment, aggregation, maturation, and dispersal MRSA: methicillin-resistant *Staphylococcus aureus*,* *EPS: extracellular polymeric substances Image Credit: Authors using PowerPoint (Microsoft Corp., Redmond, WA, USA)

Dispersed cells often exhibit increased virulence and invasiveness compared with their biofilm-associated counterparts, contributing to the progression of infection and the establishment of secondary sites of colonization (Figure 1) [9].

The MRSA biofilm matrix is a highly organized and dynamic structure composed of multiple extracellular components that collectively contribute to its stability, architecture, and resistance to host defenses and antimicrobial agents. The primary constituents include polysaccharides, proteins, eDNA, and, to a lesser extent, lipids.

Polysaccharides, particularly PIA, also known as poly-N-acetylglucosamine (PNAG), are key structural components of the biofilm matrix and are synthesized by enzymes encoded by the icaADBC operon. PIA facilitates intercellular adhesion, promotes biofilm accumulation, and contributes to immune evasion by shielding bacterial cells from phagocytosis and antimicrobial peptides [10]. Despite its importance, the reliance on PIA varies significantly among MRSA strains.

Proteins represent another major component of the MRSA biofilm matrix and play a crucial role, particularly in PIA-independent biofilms. These include MSCRAMMs such as fibronectin-binding proteins (fnbA/fnbB), clumping factors (clfA/clfB), and other adhesins that mediate attachment and intercellular interactions. Structural proteins such as Bap, Aap, and Eap further enhance cell-to-cell cohesion and biofilm integrity. Additionally, autolysins like Atl contribute both to cell lysis and the release of extracellular components essential for matrix formation.

eDNA is a critical structural and functional component of MRSA biofilms. It is primarily released through controlled autolysis and serves as a scaffold that stabilizes the biofilm matrix by interacting with proteins and polysaccharides. eDNA enhances initial adhesion, promotes biofilm maturation, and facilitates horizontal gene transfer, thereby contributing to the dissemination of antibiotic resistance genes and increased adaptability of the bacterial population [7,10].

Lipids, although present in smaller quantities, also contribute to matrix stability and may influence biofilm hydrophobicity and interactions with host surfaces. Their precise role remains less well defined but is increasingly recognized as part of the complex biofilm milieu.

MRSA exhibits significant phenotypic diversity in biofilm formation, particularly in the relative dependence on PIA. While MSSA strains more commonly rely on PIA-mediated intercellular adhesion, MRSA strains frequently form PIA-independent biofilms that are predominantly composed of proteins and eDNA. In these biofilms, surface-associated and secreted proteins, along with eDNA, compensate for reduced or absent PIA production, resulting in structurally distinct yet equally robust biofilm architectures.

This shift toward PIA-independent mechanisms in MRSA is often associated with regulatory changes involving global regulators such as agr and sarA, as well as environmental pressures, including antibiotic exposure and host immune responses. PIA-independent biofilms tend to exhibit increased resistance to enzymatic degradation targeting polysaccharides but may be more susceptible to proteases or DNases, highlighting important therapeutic considerations.

Clinically, this phenotypic heterogeneity has significant implications. Variability in matrix composition can influence diagnostic detection methods and the efficacy of anti-biofilm strategies. For example, treatments targeting polysaccharide synthesis may be less effective against protein- and eDNA-rich biofilms, necessitating combination approaches. Furthermore, the enhanced capacity for horizontal gene transfer within eDNA-rich biofilms may facilitate the spread of antibiotic resistance, contributing to persistent and recurrent infections [8].

Genotypic method: molecular regulation of MRSA biofilm formation

Biofilm formation is under tight genetic control. The major regulatory systems include the ica operon, the agr quorum-sensing system, sarA, and the alternative sigma factor sigB. Additional regulators such as ArlRS and LytSR also influence matrix production and eDNA release. The ica operon, icaADBC, encodes enzymes responsible for the synthesis of PIA, also called poly-N-acetyl glucosamine (PNAG). PIA increases intercellular cohesion and stability of the biofilm matrix. Expression of ica is modulated by icaR, a transcriptional repressor that is sensitive to environmental cues like osmolarity and glucose concentration. Importantly, although ica is the canonical mechanism, many MRSA strains form PIA‑independent biofilms that rely more on surface proteins and eDNA [1,2].

The agr quorum‑sensing system coordinates expression of virulence factors based on cell density. It produces an autoinducing peptide sensed by the agrC/agrA two-component system. When activated at high cell densities, agr upregulates secreted proteases and downregulates cell-surface adhesins, thereby promoting biofilm dispersal [3]. Thus, low agr activity promotes stable, thick biofilms, whereas high agr activity promotes biofilm dispersal. This dual role makes agr a potential therapeutic target.

sarA plays a crucial role in promoting biofilm formation by repressing extracellular protease production while enhancing the expression of surface adhesins. Through these coordinated actions, sarA supports the stability and development of biofilms. Mutations in sarA have been shown to reduce biofilm biomass and attenuate virulence, highlighting its importance as a key regulatory node in staphylococcal pathogenicity (Table 1) [4].

**Table 1:** Major genes associated with MRSA biofilm formation icaA: intercellular adhesion gene A, icaD: intercellular adhesion gene D, Bap: biofilm-associated protein, Agr: accessory gene regulator, sarA: staphylococcal accessory regulator A, fnbA/fnbB: fibronectin-binding protein A and B

Gene	Function	Clinical relevance
icaA	Polysaccharide synthesis	Biofilm accumulation
icaD	Enhances biofilm production	Increased virulence
Bap	Biofilm-associated protein	Surface adherence
Agr	Quorum-sensing regulation	Biofilm dispersal
sarA	Regulatory protein	Biofilm maturation
fnbA/fnbB	Fibronectin-binding proteins	Host tissue adhesion

The SigB system is activated under environmental stress (oxidative stress, antibiotics) and promotes expression of adhesins, aiding initial attachment and tolerance. Additional regulatory systems, such as ArlRS (autolysis control) and LytSR (eDNA release), contribute to matrix stability and structural remodeling [5].

Recent advances in the molecular regulation of MRSA biofilm formation highlight a complex interplay between structural genes, global regulators, and emerging signaling pathways. In addition to the icaADBC operon, which mediates PIA synthesis, MRSA biofilms frequently depend on adhesin-encoding genes such as fnbA/B, clfA/B, and cna, as well as autolysis-associated genes (atl, lytM) that facilitate eDNA release and matrix development [15]. Biofilm formation is tightly regulated by global regulatory systems, including agr, which promotes quorum-sensing-mediated dispersal, and sarA, which enhances biofilm accumulation, as well as stress-response regulators such as sigB [16]. Additional two-component systems, including saeRS and arlRS, further modulate adhesion, virulence, and early biofilm establishment [17]. Emerging evidence also implicates secondary signaling molecules such as cyclic di-adenosine monophosphate (c-di-AMP) and the LuxS/AI-2 quorum-sensing system in fine-tuning biofilm architecture, persistence, and antibiotic tolerance. Importantly, genotype-phenotype correlations indicate that biofilm-forming capacity is influenced by regulatory dysfunction (e.g., agr inactivity), clonal lineage variation, and environmental conditions rather than methicillin resistance alone, reinforcing the multifactorial nature of MRSA biofilm phenotypes in clinical settings.

Phenotypic detection of MRSA biofilm production

Phenotypic assays remain practical, widely used methods in clinical laboratories for screening and characterization of biofilm-producing MRSA isolates due to their simplicity and cost-effectiveness. Among these, the Congo red agar (CRA) assay is a qualitative method that differentiates slime-producing strains based on colony morphology and color, with black colonies indicating biofilm producers and red colonies indicating non-producers. Although inexpensive and easy to perform, the CRA method has limited sensitivity and may not reliably detect all biofilm-forming strains. In contrast, the microtiter plate assay is considered the gold standard for quantitative assessment of biofilm formation. In this method, bacterial isolates are cultured in microtiter wells, followed by staining of adherent cells with crystal violet and measurement of optical density at 570 nm. Based on optical density values, strains are classified as non-biofilm producers or as weak, moderate, or strong biofilm producers, enabling accurate quantification of biofilm biomass and effective phenotypic differentiation [6]. Another commonly used qualitative technique is the tube adherence method, in which bacterial cultures are incubated in glass or plastic tubes and subsequently stained to visualize biofilm formation as a visible film lining the walls and bottom of the tube. While this method is useful for preliminary screening, it lacks the precision and reproducibility of quantitative assays (Table 2).

**Table 2:** Phenotypic methods for biofilm detection

Method	Principle	Advantages	Limitations
Congo red agar	Colony morphology	Simple and inexpensive	Low sensitivity
Tube method	Adherence to tube walls	Easy screening	Subjective interpretation
Microtiter plate assay	Optical density quantification	Gold standard	Requires standardization

Clinical implications of MRSA biofilms

MRSA biofilms are strongly associated with chronic and device-related infections, including those involving catheters, prosthetic joints, and cardiac implants. Biofilm-associated cells display antibiotic tolerance up to 1,000-fold higher than that of planktonic bacteria, contributing to treatment failure and recurrence [1,2].

Biofilms also serve as reservoirs for resistant strains, promoting horizontal gene transfer of mecA and other resistance determinants [3]. Patients with indwelling devices and chronic wounds often experience prolonged hospital stays, increased morbidity, and higher healthcare costs [4].

Notably, MRSA biofilms complicate surgical interventions. Removal of infected devices is frequently required to achieve eradication, as conventional antibiotics alone are insufficient [5,6].

Antibiotic resistance in biofilms

Biofilm formation exacerbates multidrug resistance through several interconnected mechanisms. The EPS matrix restricts antibiotic penetration, limiting effective antibiotic concentrations within the biofilm. Additionally, the altered microenvironment, characterized by oxygen and nutrient gradients, reduces bacterial growth rates, thereby decreasing the efficacy of antibiotics that target actively dividing cells. Biofilms also trigger stress response pathways, in which genes such as sigB upregulate efflux pumps and DNA repair mechanisms, thereby further enhancing resistance. Moreover, the presence of persister cells, which are dormant subpopulations, enables bacteria to survive even at high antibiotic concentrations and contributes to recurrent infections. Finally, the dense structure of biofilms facilitates horizontal gene transfer, promoting the exchange of plasmids and transposons and accelerating the spread of resistance genes [3].

Emerging anti-biofilm therapeutic strategies

DNase I degrades eDNA within the biofilm matrix, while Dispersin B specifically targets and breaks down PIA polysaccharides, both of which are critical structural components of biofilms. By disrupting these key elements, these enzymes weaken the biofilm's integrity, and, when used in combination, enzymatic treatments have been shown to significantly enhance antibiotic susceptibility [10,11].

Targeting the agr and sarA regulatory systems represents an effective strategy to prevent biofilm maturation or promote biofilm dispersal. By interfering with these key quorum-sensing and regulatory pathways, it is possible to disrupt the coordinated expression of virulence factors and biofilm-associated genes. For instance, agents such as the RNAIII-inhibiting peptide and various small-molecule inhibitors have been explored for their ability to modulate these systems, thereby reducing biofilm formation and enhancing susceptibility to treatment [18].

Lytic bacteriophages that specifically target MRSA biofilms have demonstrated significant biofilm clearance in both in vitro and in vivo studies. These phages can penetrate and disrupt the biofilm structure while lysing bacterial cells. Moreover, the use of phage cocktails, which combine multiple phages with different host specificities, may help overcome biofilm heterogeneity and improve overall treatment efficacy [19].

Lipid-, polymer-, and metal-based nanoparticles have been shown to enhance antibiotic penetration into biofilms by facilitating their transport through the protective matrix. These nanocarriers can also enable controlled, targeted drug release, thereby increasing the local antibiotic concentration at the site of infection while minimizing systemic toxicity. As a result, nanoparticle-based delivery systems represent a promising approach for improving the effectiveness of antimicrobial therapies against biofilm-associated infections [20,21].

Silver, chitosan, and other nanocoatings applied to medical devices play a significant role in reducing initial bacterial attachment, thereby inhibiting the early stages of biofilm formation. By preventing microbial adhesion to device surfaces, these coatings help stop biofilm establishment before infection can occur, making them an effective preventive strategy in clinical settings.

Critical analysis

Despite substantial advances in understanding MRSA biofilm formation, several limitations and inconsistencies remain in current research. One major challenge is the lack of standardization among phenotypic biofilm detection methods. Techniques such as the CRA method, tube adherence assay, and microtiter plate assay often yield variable results due to differences in experimental conditions, interpretation criteria, and incubation parameters.

Another important limitation is the inconsistent correlation between genotypic determinants and phenotypic biofilm expression. Although genes such as icaA, icaD, bap, and fnbA are frequently associated with biofilm formation, some MRSA isolates lacking these genes still demonstrate strong biofilm-producing capacity, suggesting the involvement of alternative regulatory pathways and environmental influences.

Geographical variation in MRSA clones and resistance profiles also contributes to heterogeneity in reported findings. Many studies have small sample sizes and are limited to single-center investigations, limiting the generalizability of their conclusions. Furthermore, most currently available studies focus primarily on in vitro analyses, whereas in vivo biofilm dynamics and host-pathogen interactions remain underexplored.

The emergence of multidrug-resistant biofilm-associated MRSA strains presents a significant therapeutic challenge. Conventional antibiotics often demonstrate limited efficacy against mature biofilms due to restricted penetration, altered metabolic activity, and the presence of persister cells. Consequently, there is an urgent need for novel anti-biofilm strategies, including nanotechnology-based therapeutics, quorum-sensing inhibitors, antimicrobial peptides, bacteriophage therapy, and biofilm-disrupting agents.

Future research should prioritize standardized methodologies, multicenter genomic studies, and the development of targeted therapeutics to improve the clinical management of biofilm-associated MRSA infections.

## Conclusions

Biofilm formation represents a pivotal virulence mechanism in MRSA, significantly contributing to persistent colonization, enhanced antimicrobial tolerance, immune evasion, and the establishment of chronic and device-associated infections. The intricate regulation of biofilm development through key genetic and regulatory determinants, including icaADBC, agr, sarA, sigB, ArlRS, and LytSR, underscores the complexity of MRSA pathogenicity and highlights potential molecular targets for therapeutic intervention. Comprehensive phenotypic and genotypic characterization of biofilm-producing strains is therefore essential for accurate diagnosis, effective antimicrobial selection, infection control, and epidemiological surveillance.

In view of the increasing burden of MRSA, novel anti-biofilm strategies, such as enzymatic disruption of the biofilm matrix, bacteriophage-based therapy, quorum-sensing inhibition, nanoparticle-mediated drug delivery systems, and anti-adhesive or antimicrobial surface coatings, have emerged as promising adjuncts to conventional antimicrobial therapy. The integration of these innovative approaches with robust antimicrobial stewardship practices, early biofilm detection, and optimized medical device management may substantially improve therapeutic outcomes, reduce treatment failure, and limit the dissemination of resistant MRSA strains. Continued translational and clinical research is warranted to validate the efficacy, safety, and clinical applicability of these emerging therapeutic modalities in combating biofilm-associated MRSA infections.
